# Supplementary material for: A high-resolution transcriptomic and spatial atlas of cell types in the whole mouse brain
Source: Nature. 2023 Dec 13;624(7991):317–32. doi: 10.1038/s41586-023-06812-z (PMC10719114; doi:10.1038/s41586-023-06812-z)
Supplement: Supplementary file 1 — Reporting Summary [file 41586_2023_6812_MOESM1_ESM.pdf]

Corresponding author(s): Zizhen Yao & Hongkui Zeng
Last updated by author(s): 10/27/2023

Reporting Summary

Nature Portfolio wishes to improve the reproducibility of the work that we publish. This form provides structure for consistency and transparency in reporting. For further information on Nature Portfolio policies, see our Editorial Policies and the Editorial Policy Checklist.

Statistics

For all statistical analyses, confirm that the following items are present in the figure legend, table legend, main text, or Methods section.

n/a	Confirmed	
☐	☒	The exact sample size (*n*) for each experimental group/condition, given as a discrete number and unit of measurement
☐	☒	A statement on whether measurements were taken from distinct samples or whether the same sample was measured repeatedly
☒	☐	The statistical test(s) used AND whether they are one- or two-sided<br>*Only common tests should be described solely by name; describe more complex techniques in the Methods section.*
☒	☐	A description of all covariates tested
☒	☐	A description of any assumptions or corrections, such as tests of normality and adjustment for multiple comparisons
☐	☒	A full description of the statistical parameters including central tendency (e.g. means) or other basic estimates (e.g. regression coefficient) AND variation (e.g. standard deviation) or associated estimates of uncertainty (e.g. confidence intervals)
☒	☐	For null hypothesis testing, the test statistic (e.g. *F*, *t*, *r*) with confidence intervals, effect sizes, degrees of freedom and *P* value noted<br>*Give P values as exact values whenever suitable.*
☒	☐	For Bayesian analysis, information on the choice of priors and Markov chain Monte Carlo settings
☒	☐	For hierarchical and complex designs, identification of the appropriate level for tests and full reporting of outcomes
☒	☐	Estimates of effect sizes (e.g. Cohen's *d*, Pearson's *r*), indicating how they were calculated

Our web collection on statistics for biologists contains articles on many of the points above.

Software and code

Policy information about availability of computer code

Data collection	scRNA-seq data was collected using the 10x Genomics Chromium Single Cell 3' v2 and v3 kits and the Chromium Next GEM Single Cell Multiome ATAC + Gene Expression Reagent Bundle. Sequencing was performed on the Illumina HiSeq2500 and NovaSeq6000.<br>Flow cytometry data were acquired on a BD FACSAriaII running FACSdiva v8.<br>MERFISH data were acquired using the Vizgen MERSCOPE platform.

Data analysis

Raw scRNA-seq fastq files were processed with the CellRanger v6.0.1 pipeline. Raw 10xMultiome fastq files were processed using the 10x Genomics CellRanger Arc (v2.0) workflow with default parameters.
Doublets were identified using a modified version of the DoubletFinder algorithm which is available in scrattch.hicat (https://github.com/AllenInstitute/scrattch.hicat, v1.0.9).
scRNA-seq clustering and differential gene expression analysis was performed in R (v4.1.3) using the scrattch.bigcat package (https://github.com/AllenInstitute/scrattch.bigcat, v0.0.5), which also contains many functions to visualize the data together with the scrattch.vis package (https://github.com/AllenInstitute/scrattch.vis, v0.0210). Scrattch.bigcat adopted the parquet file format for storing sparse matrix, which allows for manipulation of matrices that are too large to fit in memory through memory mapping to files on disk. The whole gene count matrices were chunked to smaller parquet files with bin size of 50,000 for cells, and 500 for genes, which could be loaded efficiently and concurrently using the arrow package (v12.0.1, (https://github.com/apache/arrow/, https://arrow.apache.org/docs/r/). For joint clustering of 10xv2 and 10xv3 data, we used BiocNeighbor package (v1.16.0, https://github.com/LTLA/BiocNeighbors) for computing KNN using Euclidean distance within modality and Cosine distance across modality using the Annoy algorithm (v1.17.1, https://github.com/spotify/annoy).
Raw MERSCOPE data were decoded using Vizgen software (v231). Mapping of MERFISH data to the scRNASeq taxonomy was performed using the scrattch-mapping package (v0.2, https://github.com/AllenInstitute/scrattch.mapping).
To visualize the scRNA-seq data and MERFISH data we used the single-cell data visualization tool cirrocumulus (v1.1.56, https://cirrocumulus.readthedocs.io/en/latest/).

For manuscripts utilizing custom algorithms or software that are central to the research but not yet described in published literature, software must be made available to editors and reviewers. We strongly encourage code deposition in a community repository (e.g. GitHub). See the Nature Portfolio guidelines for submitting code & software for further information.

Data

Policy information about availability of data

All manuscripts must include a data availability statement. This statement should provide the following information, where applicable:

- Accession codes, unique identifiers, or web links for publicly available datasets
- A description of any restrictions on data availability
- For clinical datasets or third party data, please ensure that the statement adheres to our policy

The scRNA-seq FASTQ files were deposited in the NeMO archive and are available under accession https://assets.nemoarchive.org/dat-qg7n1b0. The MERFISH raw data are is available at Brain Image Library (BIL) under DOI https://doi.org/10.35077/g.610. Instruction for access of the processed 10X data is available at https://github.com/AllenInstitute/abc_atlas_access/blob/main/descriptions/WMB-10X.md, and instruction for access of the processed MERFISH data is available at https://github.com/AllenInstitute/abc_atlas_access/blob/main/descriptions/MERFISH-C57BL6J-638850.md

Human research participants

Policy information about studies involving human research participants and Sex and Gender in Research.

Reporting on sex and gender	N/A. This study does not involve human research participants.
Population characteristics	N/A
Recruitment	N/A
Ethics oversight	N/A

Note that full information on the approval of the study protocol must also be provided in the manuscript.

Field-specific reporting

Please select the one below that is the best fit for your research. If you are not sure, read the appropriate sections before making your selection.

☒ Life sciences ☐ Behavioural & social sciences ☐ Ecological, evolutionary & environmental sciences

For a reference copy of the document with all sections, see nature.com/documents/nr-reporting-summary-flat.pdf

Life sciences study design

All studies must disclose on these points even when the disclosure is negative.

Sample size

Sample size calculations were not performed. For scRNA-seq data, sample size was determined based on extensive prior experience using these technologies in brain. Post-hoc analysis of clustering shows that it is robust to downsampling, which indicates that the sample size is sufficient to distinguish cell types as described in the manuscript.
For MERFISH data we evenly sampled a mouse brain to capture most brain regions and get a even distribution of cells types throughout the brain. We compared the results from this mouse with those from Zhang et al companion paper and found the results are consistent.

Data exclusions

Cells from scRNA-seq data underwent stringent QC and cell quality was assessed based on gene detection, qc score, and doublet score.The qc score was calculated by summing the log transformed expression of a set of genes whose expression level is decreased significantly in poor

quality cells (full list of genes in Supplementary Table 4). We used this qc score to quantify the integrity of cytoplasmic mRNA content, which tended to show bimodal distribution. Cells at the low end were very similar to single nuclei, which we removed for downstream analysis. Doublets were identified using a modified version of the DoubletFinder algorithm172 (available in scrattch.hicat, https://github.com/AllenInstitute/scrattch.hicat, v1.0.9) and removed when doublet score > 0.3. Threshold parameters (qc score and gene counts) and number of cells filtered are summarized in Supplementary Table 4. For example, for neurons (excluding granule cells) we used gene counts cutoff of 2,000 and qc score cutoff of 200.
Post-clustering we excluded noise clusters which are clusters with significantly lower gene detection due to extensive drop out, and clusters due to doublets or contamination. We first identified doublet clusters based on the co-expression of any pair of broad class marker genes using find_doublet_by_marker function in scrattch.bigcat package. To identify other doublet clusters, we searched for triplets of clusters A, B and C, wherein A was the putative doublet cluster, such that up-regulated genes of A relative to B largely overlapped with up-regulated genes in C relative to B, and up-regulated genes in A relative to C largely overlapped with up-regulated genes of B relative to C. After removing all doublet clusters, we then identified clusters with lower gene detection. To do that, we identified pairs of clusters such that one cluster with at least 50% fewer UMIs or >100 lower QC score, smaller size, and no more than one up-regulated gene relative to another cluster was identified as the low-quality cluster.

For MERFISH data the cell-by-gene table containing segmented cells was filtered to keep cells with a volume > 100 µm3 and < 3,000 µm3, that have at least 15 genes detected and contain a minimum of 40 but no more than 3,000 mRNA molecules to remove low quality cells and doublets that are outside of these ranges.

Replication

Most of the scRNA-seq experiments were carried out at least twice independently and at least 2 mice and multiple brain dissections were used. For MERFISH, our collaborator lab generated three additional datasets with additional genes tested (see Zhang et al companion paper). All replicates have been included in the study.

Randomization

Randomization of animals to different groups is not relevant to our study design. There were no experimental vs. control groups.

Blinding

Prior to clustering, single cell transcriptomes were analyzed for previously known marker genes and were segregated into large groups: non-neuronal, glutamatergic and GABAergic. Clustering was then performed blind to the cell source or any other metadata that could reveal sample identity.

Reporting for specific materials, systems and methods

We require information from authors about some types of materials, experimental systems and methods used in many studies. Here, indicate whether each material, system or method listed is relevant to your study. If you are not sure if a list item applies to your research, read the appropriate section before selecting a response.

Materials & experimental systems

n/a	Involved in the study
☒	☐ Antibodies
☒	☐ Eukaryotic cell lines
☒	☐ Palaeontology and archaeology
☐	☒ Animals and other organisms
☒	☐ Clinical data
☒	☐ Dual use research of concern

Methods

n/a	Involved in the study
☒	☐ ChIP-seq
☐	☒ Flow cytometry
☒	☐ MRI-based neuroimaging

Animals and other research organisms

Policy information about studies involving animals; ARRIVE guidelines recommended for reporting animal research, and Sex and Gender in Research

Laboratory animals

All 317 animals used in this study were house mice (Mus musculus) maintained on the C57BL/6J background. Animals were euthanized at P53-59 (n = 141), P50-52 (n = 3), or P60-71 (n = 173). Each animal's unique ID, sex, age, and genotype are listed in Supplementary Table 2.
Mice had ad libitum access to food and water and were group-housed within a temperature- (21-22°C), humidity- (40-51%), and light- (14/10 hr light/dark cycle, or 12/12 hr reversed light/dark cycle) controlled room within the vivariums of the Allen Institute for Brain Science.

Wild animals

This study did not involve wild animals.

Reporting on sex

For each brain region, both male and female mice were used to collect scRNA-seq data. Though sex-balancing was successful at the level of brain region, after clustering of the data we identified a small number clusters that were either sex-dominant or sex-specific. These are described in the manuscript.

Field-collected samples

This study did not involve field-collected samples.

Ethics oversight

All experimental procedures related to the use of mice were approved by the Institutional Animal Care and Use Committee of the Allen Institute for Brain Science, in accordance with NIH guidelines.

Note that full information on the approval of the study protocol must also be provided in the manuscript.

Flow Cytometry

Plots

Confirm that:

- [ ] The axis labels state the marker and fluorochrome used (e.g. CD4-FITC).
- [ ] The axis scales are clearly visible. Include numbers along axes only for bottom left plot of group (a 'group' is an analysis of identical markers).
- [ ] All plots are contour plots with outliers or pseudocolor plots.
- [ ] A numerical value for number of cells or percentage (with statistics) is provided.

Methodology

Sample preparation	Sample preparation was done according to protocols: Allen Institute for Brain Science 2021. Slice Preparation with Tissue Dissociation - Mouse Protocol. protocols.io https://dx.doi.org/10.17504/protocols.io.bq6wmzfe and Allen Institute for Brain Science 2020. FACS Single Cell Sorting. protocols.io https://dx.doi.org/10.17504/protocols.io.be4cjgsw.<br><br>We used the Allen Mouse Brain Common Coordinate Framework version 3 (CCFv3; RRID: SCR_002978) ontology65 (http://atlas.brain-map.org/, Supplementary Table 1) to define brain regions for profiling and boundaries for dissection. We covered all regions of the brain using sampling at top-ontology level with judicious joining of neighboring regions (Supplementary Table 3, Extended Data Figure 1d-e). These choices were guided by the fact that microdissections of small regions were difficult. Therefore, joint dissection of neighboring regions was sometimes necessary to obtain sufficient numbers of cells for profiling. Comparison with subsequently generated MERFISH data showed that our CCF-based microdissections were largely accurate at cell subclass and major brain region levels (Extended Data Figure 2h).<br><br>Single cells were isolated by adapting previously described procedures. The brain was dissected, submerged in ACSF, embedded in 2% agarose, and sliced into 350-µm coronal sections on a compresstome (Precisionary Instruments). Block-face images were captured during slicing. Regions of interest (ROIs) were then microdissected from the slices and dissociated into single cells as previously described. Fluorescent images of each slice before and after ROI dissection were taken at the dissection microscope. These images were used to document the precise location of the ROIs using annotated coronal plates of CCFv3 as reference.<br><br>Dissected tissue pieces were digested with 30 U/ml papain (Worthington PAP2) in ACSF for 30 minutes at 30°C. Due to the short incubation period in a dry oven, we set the oven temperature to 35°C to compensate for the indirect heat exchange, with a target solution temperature of 30°C. Enzymatic digestion was quenched by exchanging the papain solution three times with quenching buffer (ACSF with 1% FBS and 0.2% BSA). Samples were incubated on ice for 5 minutes before trituration. The tissue pieces in the quenching buffer were triturated through a fire-polished pipette with 600-µm diameter opening approximately 20 times. The tissue pieces were allowed to settle and the supernatant, which now contained suspended single cells, was transferred to a new tube. Fresh quenching buffer was added to the settled tissue pieces, and trituration and supernatant transfer were repeated using 300-µm and 150-µm fire polished pipettes. The single cell suspension was passed through a 70-µm filter into a 15-ml conical tube with 500 µl of high BSA buffer (ACSF with 1% FBS and 1% BSA) at the bottom to help cushion the cells during centrifugation at 100 x g in a swinging bucket centrifuge for 10 minutes. The supernatant was discarded, and the cell pellet was resuspended in the quenching buffer. We collected 1,508,284 cells without performing FACS. The concentration of the resuspended cells was quantified, and cells were immediately loaded onto the 10x Genomics Chromium controller.<br><br>To enrich for neurons or live cells, cells were collected by fluorescence-activated cell sorting (FACS, BD Aria II running FACSdiva v8) using a 130-µm nozzle. Cells were prepared for sorting by passing the suspension through a 70-µm filter and adding Hoechst or DAPI (to a final concentration of 2 ng/ml). Sorting strategy was as previously described (Tasic et al 2018), with most cells collected using the tdTomato-positive label. 30,000 cells were sorted within 10 minutes into a tube containing 500 µl of quenching buffer. We found that sorting more cells into one tube diluted the ACSF in the collection buffer, causing cell death. We also observed decreased cell viability for longer sorts.
Instrument	FACSAria II or FACSAria Fusion
Software	FACSDiva v8
Cell population abundance	Abundance of RFP+ cell populations for 10x genomics scRNA-seq were determined by hemocytometer post-FACS.
Gating strategy	The morphology gate (SSC-A vs FSC-A) here includes all events that pass FSC threshold to allow profiling of all possible RFP+ cells. SC-FSC and SC-SSC are used to exclude doublets, and RFP+ cells are sorted from the rest of the cell based on the RFP+ DAPI- phenotype. Gating strategy for RFP+ mouse neurons with an example figure is described in more detail here: Allen Institute for Brain Science 2020. FACS Single Cell Sorting. protocols.io https://dx.doi.org/10.17504/protocols.io.be4cjgsw. Because of this, a figure exemplifying the gating strategy is not provided in the SI of the manuscript.

- [ ] Tick this box to confirm that a figure exemplifying the gating strategy is provided in the Supplementary Information.
